# Taste and Physiological Responses to Glucosinolates: Seed Predator *versus* Seed Disperser

**DOI:** 10.1371/journal.pone.0112505

**Published:** 2014-11-10

**Authors:** Michal Samuni-Blank, Ido Izhaki, Yoram Gerchman, M. Denise Dearing, William H. Karasov, Beny Trabelcy, Thea M. Edwards, Zeev Arad

**Affiliations:** 1 Department of Biology, Technion-Israel Institute of Technology, Haifa 3200003, Israel; 2 Department of Evolutionary and Environmental Biology, Faculty of Natural Sciences, University of Haifa, Haifa 3498838, Israel; 3 Department of Biology and Environment, Faculty of Natural Sciences, University of Haifa, Oranim, Tivon 3600672, Israel; 4 Department of Biology, University of Utah, Salt Lake City, UT 84103, United States of America; 5 Department of Forest and Wildlife Ecology, University of Wisconsin, Madison, WI 53706, United States of America; 6 Department of Biology, Sewanee: The University of the South, Sewanee, TN 37383, United States of America; German Institute of Human Nutrition Potsdam-Rehbruecke, Germany

## Abstract

In contrast to most other plant tissues, fleshy fruits are meant to be eaten in order to facilitate seed dispersal. Although fleshy fruits attract consumers, they may also contain toxic secondary metabolites. However, studies that link the effect of fruit toxins with seed dispersal and predation are scarce. Glucosinolates (GLSs) are a family of bitter-tasting compounds. The fleshy fruit pulp of *Ochradenus baccatus* was previously found to harbor high concentrations of GLSs, whereas the myrosinase enzyme, which breaks down GLSs to produce foul tasting chemicals, was found only in the seeds. Here we show the differential behavioral and physiological responses of three rodent species to high dose (80%) *Ochradenus’* fruits diets. *Acomys russatus,* a predator of *Ochradenus’* seeds, was the least sensitive to the taste of the fruit and the only rodent to exhibit taste-related physiological adaptations to deal with the fruits’ toxins. In contrast, *Acomys cahirinus*, an *Ochradenus* seed disperser, was more sensitive to a diet containing the hydrolyzed products of the GLSs. A third rodent (*Mus musculus*) was deterred from *Ochradenus* fruits consumption by the GLSs and their hydrolyzed products. We were able to alter *M. musculus* avoidance of whole fruit consumption by soaking *Ochradenus* fruits in a water solution containing 1% adenosine monophosphate, which blocks the bitter taste receptor in mice. The observed differential responses of these three rodent species may be due to evolutionary pressures that have enhanced or reduced their sensitivity to the taste of GLSs.

## Introduction

Fleshy fruits are commonly used by plants to facilitate seed dispersal by animals. However, as there are also seed predators, the nature of plant-animal interactions is dependent on the consumers’ fruit eating behavior. Generally, there are three ways to utilize fruits: a) Utilize only the pulp - the fleshy ripe pulp is used by plants as a reward for seed dispersing animals [1]–[3]; b) utilize only the seeds - seeds are a valuable food source for many animal species [4], being of higher nutritional quality compared to ripe fruit pulp [5]; or c) utilize the pulp and the seeds - while seed dispersers utilize only the pulp, seed predators may utilize both the pulp and the seeds [6], [7].

Although fruits are intended to facilitate seed dispersal, the ripe fruits of many plants contain secondary metabolites in toxic concentrations [1], [8]–[11]. Fruit toxins are a widespread phenomenon but the study of their function is a relatively new field in ecology and ecophysiology [12]. At least seven adaptive hypotheses have been proposed for the existence of toxins in fruit [10], [13]. The majority of these hypotheses assume that toxins have an effect, either negative or positive, on fruit consumers. For example, the Gut Retention Time Hypothesis [10], [13] assumes that fruit toxins alter gut passage rates of vertebrates, while the Directed Nutritional Benefits Hypothesis [10], [13] assumes that fruit improves the health, longevity and performance of the consumer. Furthermore, several of the adaptive hypotheses assume that fruit toxins have different effects among different consumers. The Directed Deterrence Hypothesis states that fruit toxins in ripe fruit deter seed predators, but have no or little toxic effect on seed-dispersers [10], [13]–[15]. However, few data are available for the evaluation of these hypotheses, considering that the effect of fruit toxins is tightly linked to seed dispersal and predation, and thus to plant fitness. In this paper, we are going to directly address the Directed Deterrence Hypothesis.

Like all examined plant species from the order Brassicales (which includes the Resedaceae family), *Ochradenus baccatus* contains Glucosinolates (GLSs) [16]. GLSs are a family of bitter-tasting compounds [17], [18]. Bitter taste and toxicity are often coupled traits, enabling consumers to avoid toxicity [19], [20]. However, different species may have different taste sensitivities [21]. The defensive properties of bitter, intact GLSs are further enhanced upon hydrolysis by the myrosinase enzyme, a mechanism known as the “mustard-oil bomb” [22]. The defensive properties of the mustard-oil bomb include growth depression, decreased food efficiency and lesions in the liver, pancreas and kidney [22]–[24]. In Brassicales, GLSs occur throughout the tissues of plant organs, whereas myrosinases are localized in scattered myrosin cells, which are free of GLSs [24]–[26]. Other *Ochradenus* plant organs are also protected by the mustard-oil bomb [16], [27]. In the fruits of *Ochradenus* there is a unique compartmentalization, where GLSs are found only in the pulp and the myrosinase enzyme is found only in the seeds [16]. Thus, consumers that simultaneously eat the pulp and crush the seeds will have to face the hydrolyzed products of the GLSs [16].

*Ochradenus* plants co-occur with two of the three omnivorous rodent species used in this study (*Acomys russatus* and *A. cahirinus*) that differ in their interactions with the plant. *Acomys russatus* is a seed predator that eats *Ochradenus* fruit pulp and seeds; whereas *A. cahirinus* eats the fruit pulp but expels the seeds intact [6], [16]. A third omnivorous rodent, the house mouse (*Mus musculus*), is less likely to naturally feed on *Ochradenus* fruits or seeds as it is absent from desert areas were the *Ochradenus* is found. Comparing taste sensitivities of different species of rodents, and especially sympatric species can be illuminating. For example, it is known that two out of three common mouse species that share their habitat with monarch butterflies, are sensitive to the bitter-toxic taste of the glycosides and alkaloids in the butterflies [21]. Moreover, in rodents, the T2R bitter taste receptors are found to be expressed not only in the tongue, but also in the gastrointestinal tract [28]. Rodents have also been shown to behaviorally regulate bitter-toxic food intake [29] and finally, in rodents, gut retention time may be extended in response to bitter taste and thus may also regulate bitter-toxic food intake [28].

We predicted that the two *Acomys* species would be less sensitive than *M. musculus* to the taste of GLSs and their hydrolyzed products, as *Acomys* encounter and consume *Ochradenus* fruits in their natural habitat [6], [7], [16]. In this study we used high dose (80% of the diet), short-term, feeding trials. We also predicted that within a few days, we should be able to observe the negative impact of a high dose of pulp or mashed fruit diets in all three rodents. As intact GLSs are generally non-toxic, we predicted that seed disposers, like *A. cahirinus*, should not be deterred by the taste of intact GLSs in the pulp, but would keep away from pulp and seed co-consumption to avoid the mustard-oil bomb physiological consequences. As different species may have different taste sensitivities [21], it is plausible that the seed predator, *A. russatus,* has a higher perception threshold (i.e., lower sensitivity) to the taste of GLSs and their hydrolyzed products. Thus, it should be able to overcome the taste and physiological effects of intact GLSs as well as GLS-myrosinase combination. A new introduced species, like *M. musculus,* should reject intact as well as hydrolyzed GLSs.

We used adenosine monophosphate (AMP), known to block bitter taste receptors in mice [30], and monitored the rodents’ feeding behavior. We also used diets that contained intact GLSs and activated GLSs and monitored the rodents’ body mass, food intake, digestibility and gut retention time (as bitter compounds can extend retention time). We measured estrogenic activity in *Ochradenus* fruit pulp and seeds as some phytoestrogens are known for their bitter taste [18]. In addition, estrogenicity in the fruits may affect rodent metabolism and weight gain [31].

## Materials and Methods

### Ethics Statement

The experimental protocols were approved by the University of Haifa Committee of Animal Experimentation (Permit number 096/08). No specific permissions were required for the collection of fruits, as they were not collected from protected areas. The plant, *Ochradenus baccatus*, is not an endangered or protected species.

### Estrogenic Activity in Fruits

For measurement of phytoestrogen activity in *Ochradenus* fruit, pulp and seeds, we collected 200 ripe fruits in early March 2013 from five individual plants growing wild in Almog junction (31°48′N, 35°27′E). Fruits were kept at 4°C and pulp was manually separated from the seeds within one day of collection. Samples were dried for 24 h at 40°C and stored at −20°C (pulps) or room temperature (seeds). Combined pulps and combined seeds from each plant (*n* = 5 per organ type; 0.3–0.5 g dry tissue per sample) were ground using a mortar and pestle and double extracted in 55% ethanol. Samples were reconstituted in 55% ethanol to a final concentration that represented 2.7 g dry weight/ml for pulps and 3.0 g dry weight/ml for seeds (equivalent to 13.3 and 3.1 g fresh weight/ml for pulps and seeds, respectively). Dilution curves to measure estrogenic activity of pulp and seed extracts were compared to estradiol and soybean leaf extracts using transgenic *Saccharomyces cerevisiae* (baker’s yeast) expressing human estrogen receptor β (ESR2) linked to a β-galactosidase reporter gene (lacZ) [32]. Dilution curves ranged from 0.3–82 mg fresh weight per well for pulps and 0.14–19.4 mg fresh weight per well for seeds. Estrogenicity was expressed as pg estradiol equivalents per mg fresh weight.

### Study System

General information on the study system including a description of the plant, fruit collection, fruit concentration of GLSs in the pulp and myrosinase activity in the seeds and animal maintenance can be found in Samuni-Blank *et al.* [7], [16].

### Fruit Taste - Behavior

Animals (*Acomys cahirinus n* = 8; body mass = 55.0±4.4 g SE; *Acomys russatus n* = 8; body mass = 59.3±2.5 g SE; *Mus musculus n* = 8; body mass = 25.8±0.9 g SE) were placed in separate cages with 5 intact *Ochradenus* fruits (‘natural’) over-night. After 24 h the cage floor was examined for intact fruits or fruit parts (pulp or seeds). We counted the intact fruits and documented whether the mice ate only the pulp, only the seeds or both. Then, we gave the same individuals five fruits from the same batch that were soaked for ten minutes in distilled water containing 1% adenosine monophosphate (AMP), known to block bitter taste receptors in mice [30]. Remains of AMP-treated fruits were categorized after 24 h as mentioned above. Throughout the experiments, carrots, rodent chow and dog chow were provided *ad libitum*.

### Fruit Taste - Physiology

To examine the effect of taste of a mashed fruit diet (fruit pulp and seeds mashed together, causing hydrolysis of GLSs) *versus* a pulp diet (no seeds, therefore containing intact GLSs), we performed feeding trials and monitored the effects of the different diets on body mass, dry matter (DM) food intake and DM digestibility. We used adult males of: *A. cahirinus* (body mass = 50.9±1.2 g SE; *n* = 117), *A. russatus* (body mass = 53.8±1.1 g SE; *n* = 110) and wild house mouse (*M. musculus*; body mass = 29.6±0.7 g SE; *n* = 22) from captive breeding colonies maintained at the Department of Biology and Environment at the University of Haifa, Oranim.

The rodents were fed for two days with one of three possible diets (treatments): pulp (containing intact GLSs), mash (containing hydrolyzed products of GLSs) and a control (rodent chow) diet (n = 6–8 individuals per treatment). In each case the diet treatments contained on a wet mass basis: 20% rodent chow (Koffolk serial no. 19510, Tel Aviv, Israel) combined with 80% pulp (pulp was manually separated from the seeds) or mash (pulp and seeds crushed together). The control diet contained 70% rodent chow combined with 30% tap water. The total calories available in the pulp, mash and control diet was analyzed by Parr microbomb calorimeter with a benzoic acid standard.

A body mass loss of 15% was defined as the limit of tolerance. During the experiment we took daily measurements of body mass and food intake. Excreta and food leftovers were collected every day from the plastic cage floor, weighed, dried (at 50°C for 24 h) and stored at room temperature. The dry matter (DM) digestibility of food consumed by an animal was calculated from its DM intake and fecal DM output as: DM digestibility = (DM intake – fecal DM output)/DM intake.

### Gut Retention Time

To examine the effect of *Ochradenus* fruits on the *Acomys* gut retention time, we used captive adult males and females of *A. russatus* (body mass = 58.2±2.2 g SE; *n* = 25) and *A. cahirinus* (body mass = 49.3±1.6 g SE; *n* = 25). Animals were fed according to one of the four following treatments: 1) ‘Control’ - rodent chow (Koffolk serial no. 19510) homogenized with 70% tap water; 2) ‘Pulp’ – pulp, which contain the intact GLSs, manually separated from fruits. 3) ‘Seeds’ – seeds, which contain the myrosinase enzyme, manually separated from fruits and homogenized with 80% water. 4) ‘Mash’ - pulp and seeds mashed together, contain the hydrolyzed products of GLSs. Food treatment was orally administrated using 10 ml syringe into the animal mouth. All other food was denied from the animals an hour before and an hour after feeding. Orally administrated food was homogenized with 1% of inert blue plastic marker pieces that can pass through a 40 mesh screen. Fecal samples were collected every 15 min (coprophagy was not prevented), each sample was mashed in water and examined until the first markers appeared in the feces.

### Statistical Analyses

To test for differences in consumption with or without AMP, that did not fulfill normality criteria (Kolmogorov-Simirnov test), we used the Wilcoxon Signed Ranks Test. All other variables tested fulfilled normality criteria. Therefore, to compare species and treatments we used two-way ANOVA and to test for differences within each species we performed a one-way ANOVA followed by Bonferroni multiple comparisons. In all cases, significance level was set at *P*<0.05. All data are reported as means ± standard error (SE). Statistical analyses were conducted using SPSS 19.0 (SPSS, USA).

## Results

For phytoestrogen activity in pulp, we tested extracts that represented 2–510 mg fresh weight/ml (equivalent to 0.4–102 mg dry weight/ml). For phytoestrogen activity in seeds, we tested extracts that represented 1–120 mg fresh weight/ml (equivalent to 0.96–115 mg dry weight/ml). No phytoestrogenic activity was found in *Ochradenus* fruits (pulp or seeds).

### Fruit Taste - Behavior

A clear and significant difference was apparent between the two *Acomys* species in the number of seeds left intact. The seed predator, *A. russatus,* left 0.13±0.13 and 0.25±0.25 intact seeds, while the seed disperser, *A. cahirinus,* left 16.14±3.42 and 15.75±2.91 intact seeds on the non-treated and AMP treated fruits respectively. For each of the two *Acomys* species, there were no significant differences (*P*>0.05) in the number of fruits eaten and seeds left intact in the natural fruits versus the AMP-treated fruits (Table 1).

**Table 1:** Average number (± S.E.) of intact fruits (Natural) and AMP-treated fruits (AMP) fruits after 24 h by the seed predator, *A. russatus,* the seed disperser, *A. cahirinus* and a naïve rodent, *M. musculus* (*n* = 8 for each species in each of the treatments).

	Fruit left	
	Natural	AMP
**Predator^N.S^**	0.0±0.0	0.63±0.63
**Disperser^N.S^**	1.25±0.62	0.75±0.31
***M*** **. ** ***musculus***	3.8±0.72^A^	0. 5±0.5^B^

Different letters adjacent to means indicate significant difference (Wilcoxon Signed Ranks Test, *P*>0.05) among means. N.S., not significant.

For *M. musculus,* there was a significant difference between ingestion of natural and AMP treated fruits with *M. musculus* avoiding ingestion of natural fruits but consuming AMP treated fruits (*Z* = 2.26, *n* = 8, *P* = 0.024; Table 1). For non-treated fruits, five of eight *M. musculus* individuals left the natural fruits intact, two individuals ate only the seeds and one ate one whole fruit (pulp and seeds) out of five offered. As a results there were no intact seeds left separated from the pulp in the cages (seeds were either all consumed or were left inside the whole fruit). For fruits soaked with AMP, only one individual left intact fruits. The other seven individuals ate both pulp and seeds leaving 5.25±1.41 intact seeds separated from the pulp. Altogether, significantly more AMP-treated pulp and AMP-treated seeds were consumed compared to the natural pulp and seeds (*Z* = 2.45, *n* = 8, *P* = 0.014; *Z* = 2.24, *n* = 8, *P* = 0.025; respectively).

### Fruit Taste - Physiology

The total calories available in the pulp, mash and control diet were 4060±44, 4433±80 and 4154±35 cal/g, respectively.

The experiment for all *M. musculus* individuals on mash diet was terminated after one day, due to loss of 15% of body mass on mash diet.

#### Body mass

**Figure 1:**
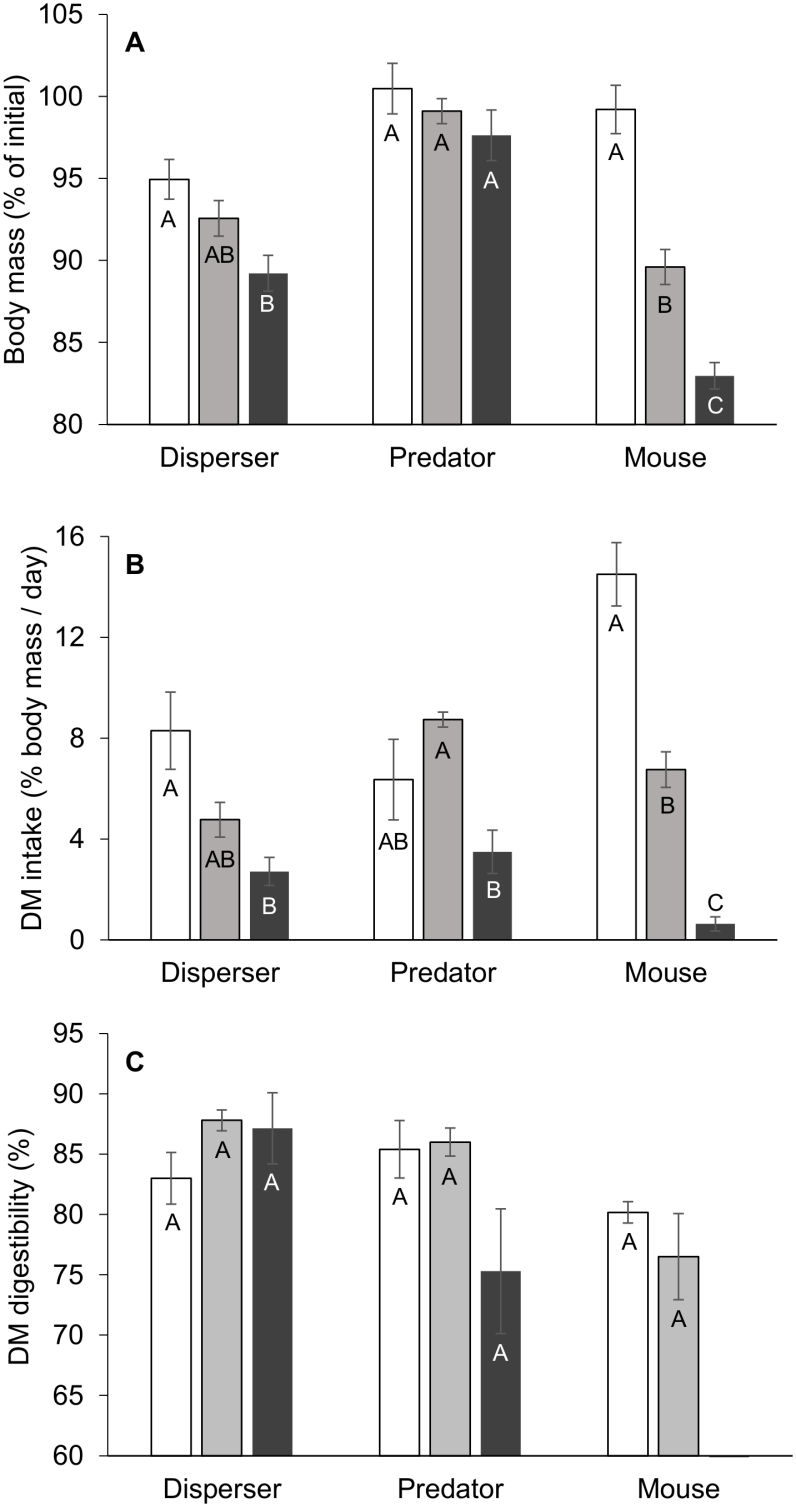
Feeding trials. Control (white bars), 80% *Ochradenus* pulp (gray bars) and 80% *Ochradenus* mash (black bars) diet of *A. cahirinus* (disperser) and *A. russatus* (predator) after two days and of *M. musculus* (mouse) after one day. *n* = 6–8 for each diet within each species. A. Body mass (% of initial). B. Dry matter intake (% body mass/day). C. Dry matter digestibility (%). Within each species, different letters at the top of the columns indicate significant differences (Bonferroni Multiple Comparison, *P*<0.05). Data are presented as means ± SE.

There were significant differences in body mass changes among species (*F*_2,54_ = 36.9, *P*<0.0001) and among treatments (*F*_2,54_ = 33.2, *P*<0.0001), as well as a significant treatment*species interaction (*F*_4,54_ = 8.3, *P*<0.0001). *M. musculus* significantly lost body mass on both pulp and mashed diets after only 24 h, while in the two *Acomys,* body mass was maintained on the control and pulp diets (Fig. 1A). All individuals recovered and returned to their original body mass a few days after the experiment ended.

#### Food intake

A significant difference in the normalized DM intake (% body mass/day) was found among treatments (*F*_2,54_ = 35.5, *P*<0.0001) and there was no main effect of species on DM intake (*F*_2,54_ = 2.7, *P* = 0.08). However, there was a significant interaction between species and treatments (*F*_4,54_ = 9.2, *P*<0.0001); *Acomys russatus* maintained DM intake on the pulp diet, while the same diet depressed DM intake of *M. musculus* (Fig. 1B).

#### Digestibility

There was a significant difference in the DM digestibility among species (*F*_2,49_ = 6.4, *P*<0.005) and there were no significant differences in DM digestibility among treatments (*F*_2,49_ = 1.6, NS). There was a significant interaction between species and treatments (*F*_3,49_ = 3.0, *P*<0.05); the DM digestibility in *A. russatus,* but not in *A. cahirinus*, decreased on the mash diet (Fig. 1C).

### Gut Retention Time

**Figure 2:**
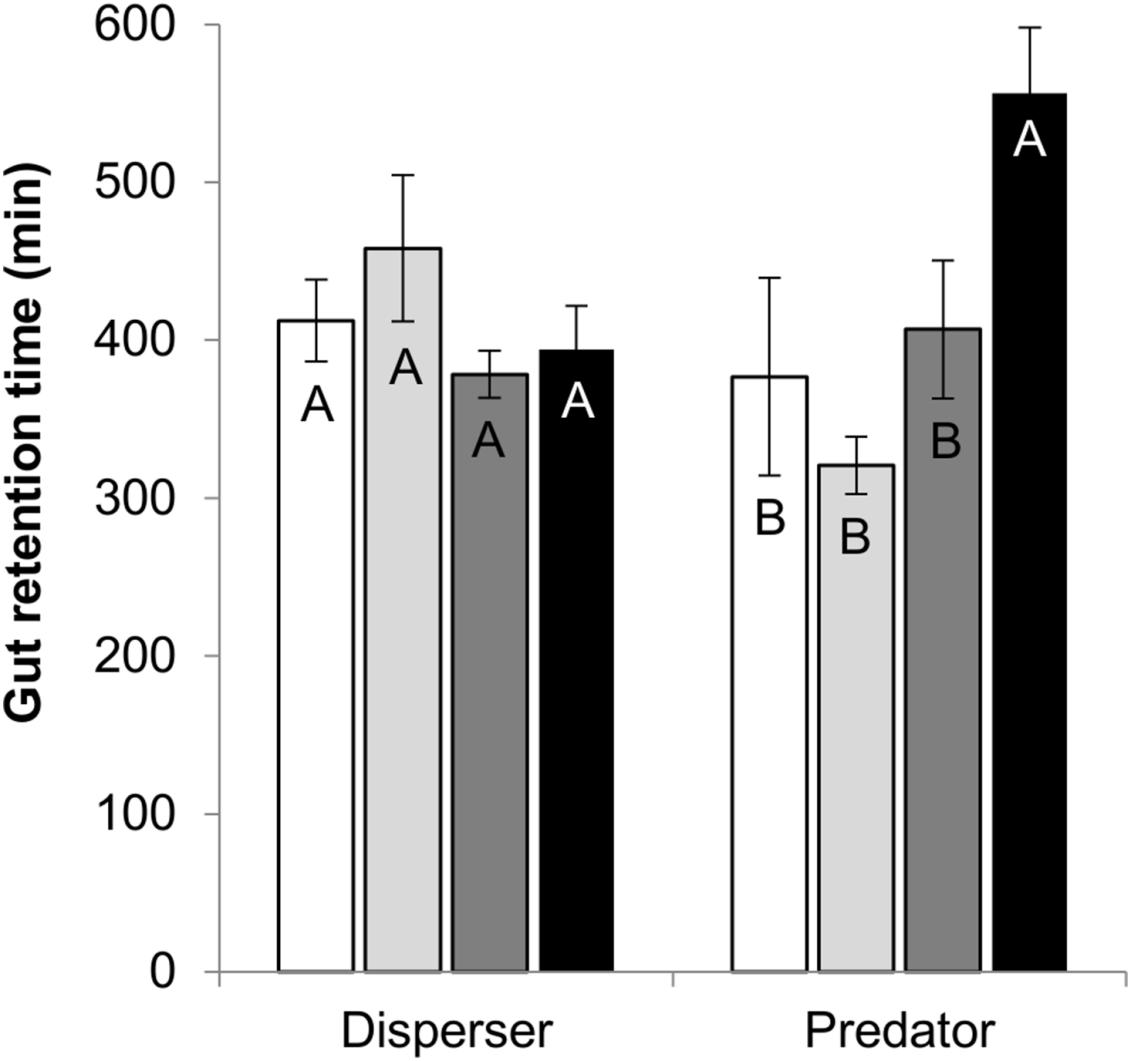
Gut retention time. Gut retention time in *Acomys cahirinus* (seed disperser) and *A. russatus* (seed predator; *n* = 6–8 for each group within each species) fed with a control diet (white bars), with *Ochradenus* pulp (light-gray bars), with *Ochradenus* seeds (dark-gray bars) or with mashed *Ochradenus* fruits (black bars). Within each species, different letters at the top of the columns indicate significant differences (Bonferroni Multiple Comparison, *P*<0.05). Data are presented as means ± SE.

There were significant differences in gut retention time among treatments (*F*_3,40_ = 3.4, *P*<0.05) but no main effect of species (*F*_1,40_ = 0.0, NS). However, there was a significant treatment*species interaction (*F*_3,40_ = 7.7, *P*<0.0001); the mash treatment significantly increased the gut retention time in *A. russatus* but not in *A. cahirinus*. Average retention time for all groups, except for *A. russatus* mash treatment, was less than eight hours (Fig. 2).

## Discussion

Previously [6], [7], [16], we revealed divergent behavioral and physiological strategies and adaptations in two coexisting species of desert rodents, *A. cahirinus* and *A. russatus,* dealing with fleshy fruit toxins. Here we demonstrate that these different strategies are probably mediated by taste.

The *Ochradenus* GLSs are likely to be an important factor limiting fruits consumption by rodents. GLSs and their hydrolyzed products are known for the deterrent quality of their bitter taste [18]. The bitter taste of the fruits that originate from GLSs in the pulp appears to be the cause of *M. musculus*’ fruit avoidance, as we were able to alter *M. musculus* behavior by using AMP. Even though *M. musculus* was first exposed to the natural fruits and only later to the AMP treated fruits, there was no evidence of learning effect as it consumed significantly more AMP treated fruits. We cannot rule out neophobia as a possible explanation to the fact that 5 out of 8 *M. musculus* did not sample the untreated fruits, since we first gave the mice untreated fruits and only later the AMP treated fruits. However, over the years, we have observed numerous first-time encounters of rodents with *Ochradenus* fruits; some rodents hold the fruit in their mouth and spit it out intact. Thus, we believe that it is more likely that the mice avoided the fruits because of their taste and not because of neophobia.

From the results we conclude that the seed predator, *A. russatus,* has as a higher perception threshold (i.e., lower sensitivity) to the taste of GLSs and their hydrolyzed products; after two days in the feeding trial *A. russatus* was the only species that consistently ate the whole fruit and maintained body mass on 80% pulp and mash diets. Also, it was the only species to have higher DM food intake on the pulp diet compared to the control diet. The differences between species are consistent with our previous work, where we have shown that after four days in the feeding trial on 50% mash diet, *A. russatus* maintained ∼90% of its initial body mass, while the seed disperser, *A. cahirinus,* maintained only ∼80% of its initial body mass [7]. In addition, on the mash treatment, *A. russatus,* but not *A. cahirinus,* significantly extended its gut retention time and decreased its DM digestibility. These physiological responses may provide more time for the operation of detoxification enzymes in the gut and liver, decrease the concentrations and slow the rate of hydrolyzed products entering the systemic circulation [28]. We suggest that these differential responses of the two species to *Ochradenus* fruits have stemmed from interspecific competition.

Another observation that emerges from comparison to [7] is that *A. russatus* performs better on high-concentration, short-term diet (80% mashed fruit, two days) than on low-concentration, long-term mash diet (one day at 25% followed by three days at 50%). Likewise, compared to the pulp diet, DM intake of *A. russatus* is lower by ∼50% on a lower concentration of mash in our previous study [7], while the difference in this study was not significant. This could be evidence of adaptive physiological mechanisms such as detoxification enzymes that *A. russatus* can employ when toxicity levels increase. Another explanation is that post-ingestional effects are not as immediate as taste and thus are less pronounced in short term trials. A third hypothesis is that the high concentration of mash increases the likelihood of *A. russatus* using torpor to conserve energy [33].

Like the *Acomys, M. musculus* exhibited a higher tolerance level (in terms of defending body mass) when feeding on the pulp diet than on the mash diet, despite its avoidance of the GLSs. Although mash diet has the highest energetic value, after only one day on this diet, *M. musculus* lost ∼15% of its initial body mass. Individuals of *M. musculus* preferred to face rapid and life threatening loss of body mass, rather than to feed on the mash diet. *Mus musculus* also significantly reduced DM food intake when feeding on the pulp diet. However, *M. musculus* consumed significantly more food on the pulp diet than on the mash diet, suggesting a preference of intact GLSs over their toxic hydrolyzed products.

This study, provides some evaluation to the existence of toxins in fruit adaptive hypotheses. In agreement with The Directed-Deterrence Hypothesis [13], *A. cahirinus* and *M. musculus* showed highest loss of mass while on mash diet. These findings also extend The Directed-Deterrence Hypothesis to the intraspecific level, in addition to the interspecific level, as they also performed better on seed dispersers’ diet (pulp) than on seed predators’ diet (mash). However, other findings of our work are in contradiction with The Directed-Deterrence Hypothesis, as they demonstrate the ability of *A. russatus* to consume the whole fruit of *Ochradenus* while exhibiting behavioral and physiological traits that allow it to avoid the negative effects of GLSs hydrolyzed products (see also [6], [7]). Moreover, our previous study demonstrated the ability of another seed predator, *A. minous*, to circumvent the activation of the GSLs by making a hole in the pulp and consuming only the seeds [7]. Our results are ambiguous also with respect to The Gut Retention Time Hypothesis, which states that fruit secondary metabolites act to alter seed passage rates; *Ochradenus baccatus* fruit toxins did alter gut passage in *A. russatus,* but not in *A. cahirinus*. This study does not provide strong support for either of the hypotheses, probably due to the fact that the two species (*A. cahirinus* and *A. russatus*) are closely related. This fact, however, is what makes this study system particularly fascinating, as it allows to study the function of fruits’ toxins in finer detail.

In the present study, we revealed the ability of *Ochradenus* plants to deter seed predators from co-consumption of pulp and seeds. Our results demonstrate that the mustard oil bomb mechanism in *Ochradenus* fruits shapes the behavioral responses of its consumers at an ecological timescale. This is also supported by our previous studies, which show, for example, that the evolutionary naïve *A. minous* was able to behaviorally avoid the GLSs-myrosinase combination in *Ochradenus* fruits. The bitter taste of the GLSs is a first line of defense against naïve rodents. The bitter taste of the hydrolyzed products of GLSs is a second barrier. Finally, seed predators that co-consume the pulp and seeds will encounter the toxic products of the GLSs. Nevertheless, the value of fleshy fruits cannot be overestimated in the desert ecosystem; thus, there is an evolutionary pressure on consumers to develop behavioral and physiological adaptations, in order to extract the greatest benefit. In this evolutionary arms race between the plant and its consumers, one will always lag behind.
